# Human embryonic and induced pluripotent stem cells maintain phenotype but alter their metabolism after exposure to ROCK inhibitor

**DOI:** 10.1038/srep42138

**Published:** 2017-02-06

**Authors:** Spyros I. Vernardis, Konstantinos Terzoudis, Nicki Panoskaltsis, Athanasios Mantalaris

**Affiliations:** 1 Biological Systems Engineering Laboratory, Department of Chemical Engineering, Imperial College London, UK; 2 Department of Haematology, Imperial College, London, UK

## Abstract

Human pluripotent stem cells (hPSCs) are adhesion-dependent cells that require cultivation in colonies to maintain growth and pluripotency. Robust differentiation protocols necessitate single cell cultures that are achieved by use of ROCK (Rho kinase) inhibitors. ROCK inhibition enables maintenance of stem cell phenotype; its effects on metabolism are unknown. hPSCs were exposed to 10 μM ROCK inhibitor for varying exposure times. Pluripotency (TRA-1-81, SSEA3, OCT4, NANOG, SOX2) remained unaffected, until after prolonged exposure (96 hrs). Gas chromatography–mass spectrometry metabolomics analysis identified differences between ROCK-treated and untreated cells as early as 12 hrs. Exposure for 48 hours resulted in reduction in glycolysis, glutaminolysis, the citric acid (TCA) cycle as well as the amino acids pools, suggesting the adaptation of the cells to the new culture conditions, which was also reflected by the expression of the metabolic regulators, *mTORC1* and *tp53* and correlated with cellular proliferation status. While gene expression and protein levels did not reveal any changes in the physiology of the cells, metabolomics revealed the fluctuating state of the metabolism. The above highlight the usefulness of metabolomics in providing accurate and sensitive information on cellular physiological status, which could lead to the development of robust and optimal stem cell bioprocesses.

Human pluripotent stem cells have the ability to self-renew and differentiate to any cell type of the body, which renders them important to both research and potential regenerative medicine therapeutic applications^1^. hPSCs, both embryonic and induced pluripotent, are optimally maintained in colonies yet efficient and robust differentiation protocols are best achieved by single cell cultures^2^. Addressing challenges of directed differentiation *in vitro*, such as scaling-up of the cultures, elimination of xeno-components, efficiency, etc., as well as understanding the epigenetic differences on methylation patterns between hESCs and hiPSCs^3^, requires precise and sensitive monitoring of cell cultures that provides insightful information on cellular state.

Metabolism is the set of life-sustaining chemical transformations within the cells that is considered as the most dynamic. It directly reacts with the environment and provides fast response to environmental perturbations. Metabolism is simultaneously both autonomous and inter-connected with the other levels of the cellular function, such as protein and gene expression as well epigenetic changes. Emerging evidence suggests that pluripotent stem cells are metabolically distinct from their differentiated counterparts and that these metabolic properties are important for stem cell identity. Furthermore, molecular regulators of energy metabolism have essential roles in stem cell fate, in particular, the decision to self-renew or differentiate and stem cells respond to fluctuations in energy states *in vivo*^4^. Specifically, metabolic activity and metabolite pools affect stem cell epigenetic mechanisms in a direct way^5^ influencing stem cell differentiation potential^6^,^7^,^8^. It is of great importance to define cell state - undifferentiated or differentiated - by its metabolic properties as this is in direct correlation with the cells’ potential.

Single cell cultures in suspension of hPSCs undergo apoptosis despite the use of culture conditions conducive to stem cell maintenance^9^,^10^. This problem has been addressed with the inhibition of ROCK (Rho-associated protein kinase), a serine-threonine kinase that phosphorylates and activates the myosin II pathway^11^,^12^,^13^,^14^,^15^,^16^ resulting in the maintenance of the differentiation potential for up to 72 hours, which is considered to be primarily mediated via the inhibition of an E-cadherin-dependent apoptotic pathway^17^,^18^,^19^,^20^. The most effective ROCK inhibitor is Y-27632^21^. hPSCs colonies, both hESCs and hiPSCs, are most commonly treated with 10 μM of Y-27632 prior to dissociation of the colonies into single cells^22^,^23^,^24^. With this protocol, single cell survival is maintained for up to 3 weeks and stem cell phenotype along with differentiation capability into all lineages are sustained^11^. Until now, it has been assumed that ROCK inhibition does not affect physiology since the hPSCs retain their stemness and survive^25^.

Herein, we have undertaken an in-depth assessment of the effect of ROCK inhibitor (Y-27632) on the dynamic metabolism of hPSCs over a 96-hour culture period^5^,^26^,^27^. Whereas no differences were observed in the pluripotency and viability of hESCs and hiPSCs on gene and protein expression levels, differences in metabolism were detected. Specifically, metabolomics analysis was able to detect changes on the metabolic physiology of the cells as early as 12 h of ROCK inhibitor treatment. Existing metabolic switches on hESCs and hiPSCs were similar on both cell types up to 48 h of exposure to Y-27632, with 24 h exposure being identified to be critical as a turning point in metabolism. Correlating with these metabolic changes, a differential expression of the metabolic regulators, p53^28^,^29^ and mTORC1^4^,^30^, was observed. The above indicate a dynamic process of adaptation of the cells to the altered environment, which is mostly projected to the metabolic level.

## Results

### hPSCs maintain pluripotency phenotype up to 48 h of exposure to ROCK inhibitor

**Figure 1:**
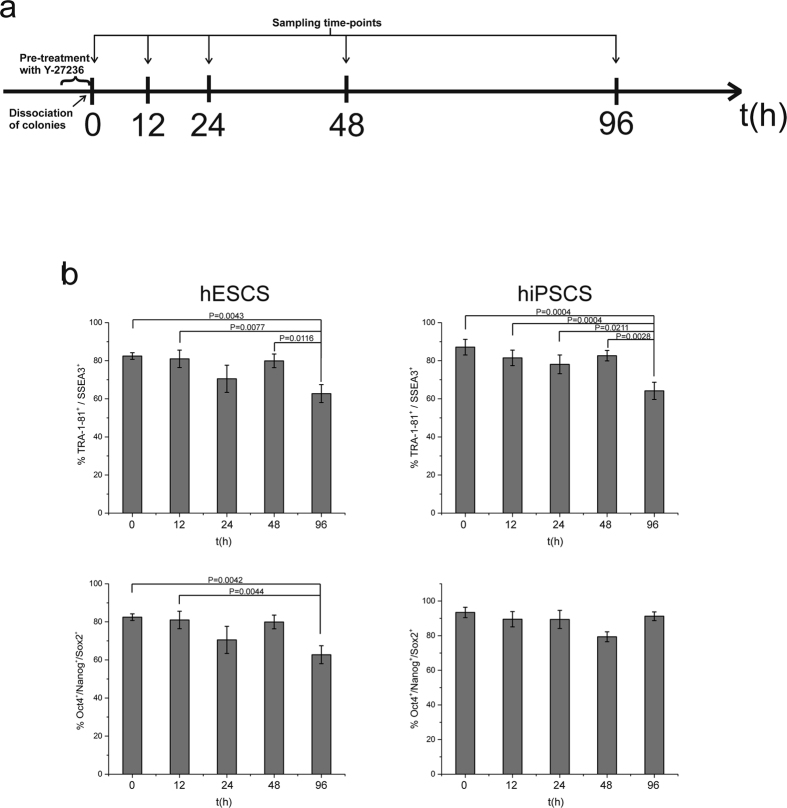
Assessment of pluripotency phenotype by conventional markers following exposure to ROCK inhibitor. (**a**) Experimental design. hPSCs were pre-treated with 10 μΜ Y-27236 ROCK inhibitor for 1–2 hours. Then, colonies were dissociated into single cells and exposed to 10 μΜ Y-27236 ROCK inhibitor. Samples were collected at five time-points: 0 h were untreated with cells remained in colonies whereas single cells were sampled at all other time-points (12 h, 24 h, 48 h and 96 h). (**b**) hPSC cell phenotype by flow cytometry and qRT-PCR was maintained for at least 48 hours following exposure to ROCK inhibitor, with reduction in stemness phenotype noted by 96 hours. *p < 0.05.

The effects of Y-27632 exposure on both hESCs and hiPSCs were evaluated by assessing gene and protein expression of pluripotency markers. Specifically, TRA-1-81 and SSEA3 expression indicated that the cells retained these pluripotency markers up to 48 h of exposure, whereas there was a reduction at 96 h in both cell types (p < 0.05; Fig. 1b). Intracellular expression of Nanog, Oct4 and Sox2 indicated maintenance of stemness at high levels (over 67% for hESCs and 80% for hiPSCs), which is reduced at 96 h exposure for hESCs (p < 0.05; Fig. 1b). *Nanog, Oct4* and *Sox2* expression did not differ at any time-point evaluated (data not shown). Consequently, gene and protein expression showed that the pluripotency phenotype remained constant for at least 48 hours after ROCK exposure and was similar to the untreated control. In all cases, cell viability was high (data not shown). Immunostaining for Oct4 and Sox2 confirmed the maintenance of pluripotency, apart from the 96 h time-point in hESCs when the staining appeared to be weaker (Supplementary Figure 1), in agreement with the flow cytometry results (Fig. 1b).

### Metabolomics analysis reveals changes in metabolism

**Figure 2:**
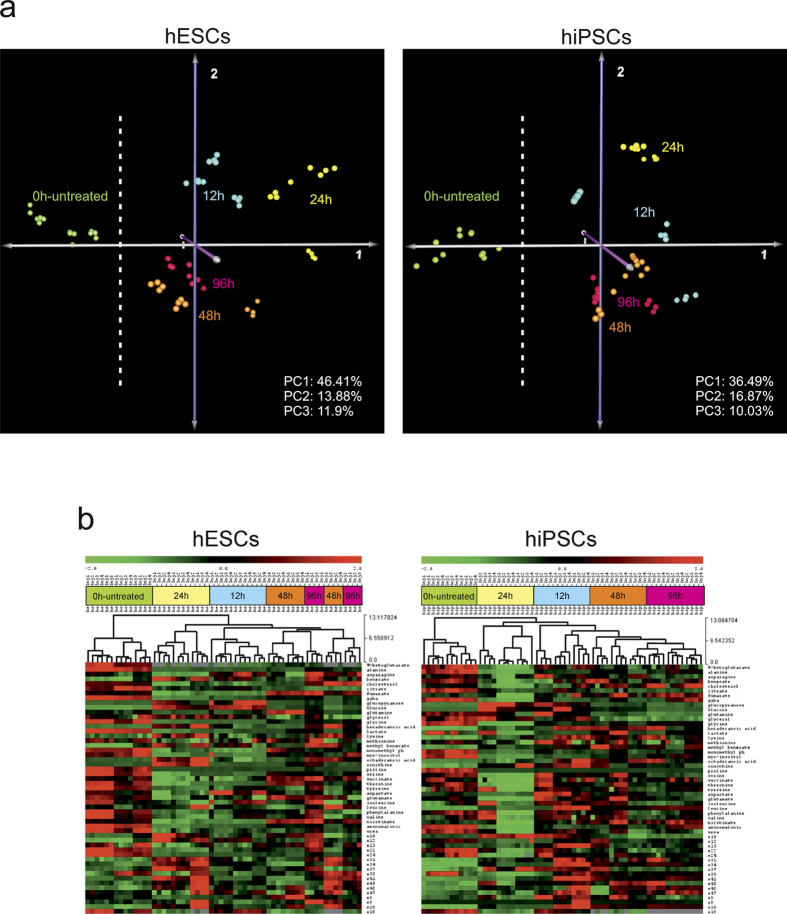
Principal Component Analysis (PCA) and Hierarchical Clustering (HCl). (**a**) PCA graphs show the difference of 0 h-untreated profiles of both hESCs and hiPSCs compared with that of the other time-points. Interestingly, profiles of the 0 h (green) for both hPSCs are grouped in the left side of the graphs. It can also be observed that 48 h and 96 h profiles are grouped together and seems closer compared to the highest spreading of 12 h and 24 h profiles. ROCK inhibitor changed metabolic physiology compared with that of the 0 h untreated cells. (**b**) HCL and heatmaps indicate that clustering of metabolic profiles is similar for both types of hPSCs and dependent on exposure time to ROCK inhibitor. Metabolism over time appear similar for both cell types throughout the experimental process, though not identical. 0 h-untreated profiles form a discrete cluster in both types of cells. (Distance Metric: Euclidean on standardized relative peak areas – stRPAs).

Multivariate statistical analysis revealed that 0 h–untreated cells (grown in colonies and not exposed to Y-27632) had discrete metabolism compared to those exposed to Y-27632 as early as 12 hrs, as highlighted by Hierarchical Clustering (HCL) analyses and heatmaps (Fig. 2b). Grouping of the metabolic profiles from cells exposed 48 h and 96 h to ROCK inhibitor demonstrated that prolonged exposure resulted in adapted cellular physiology not as discrete as that of cells exposed for 12 h and 24 h, as elucidated by the grouping in Principal Components Analysis (PCA) graphs (Fig. 2a). Alas, the distinct grouping of the hESCs and hiPSCs at 12 h exposure indicates a different physiological response of the cells (Fig. 2a).

**Figure 3:**
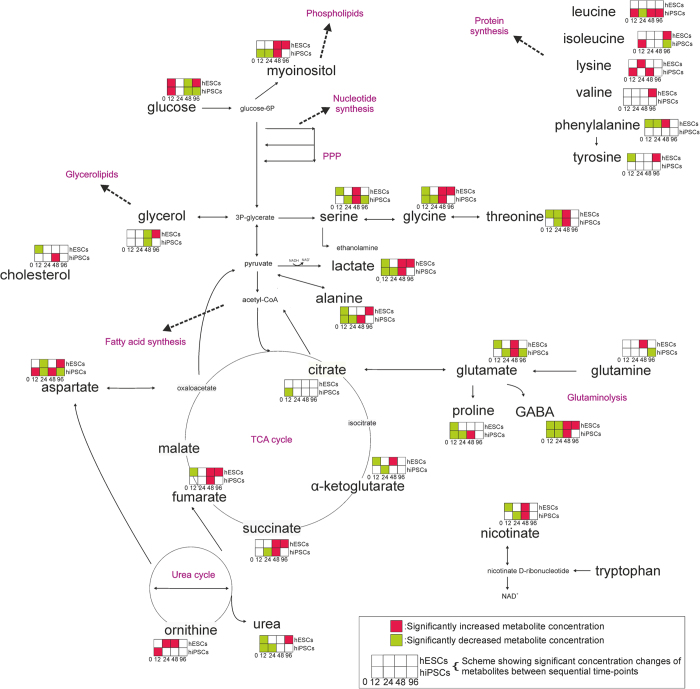
Network representation of significant metabolic changes over time. Significance analysis of microarrays (SAM) was applied between time-points. The metabolic transitions are similar up to 48 h. 48 h to 96 h transition is different between hESCs and hiPSCs. Glycolysis, glutaminolysis and amino acid pools are up-regulated at 0 h, they reach their lower level at 24 h and they increase their activity again at 48 h. At 96 h the metabolite pools are increasing for the hESCs and decreasing for the hiPSCs.

To further understand how cellular physiology changed with culture time, an in-depth assessment of the metabolic transitions was undertaken: consecutively from 0 h to 12 h, 12 h to 24 h, 24 h to 48 h and 48 h to 96 h by Significance Analysis of Microarrays (SAM) (Fig. 3). At 12 hours of Y-27632 exposure, the metabolic profiles were similar for both hESCs and hiPSCs; glucose concentration increased whereas lactate and alanine production decreased, indicating down-regulation of the glycolytic route. TCA cycle and glutaminolysis were also down-regulated. Some amino acids (glycine, proline, and GABA for both ESCs and iPSCs, and threonine, serine, phenylalanine, tyrosine for hESCs) and urea (for both cell types) were reduced. From 12 h to 24 h, the most important difference was the reduction of metabolites in the serine-glycine-threonine pathway. In addition, aspartate was reduced in both hiPSCs and hESCs. The further reduction at 24 h in the metabolite pools is more intense in hiPSCs. After 48 h of exposure time, metabolism increased as indicated by the activation of glycolysis, glutaminolysis, TCA cycle and amino acid pools (serine, glycine, threonine for both types of cells, ornithine, phenylalanine for hESCs, and aspartate, leucine, valine for hiPSCs). Overall, the metabolic behaviour of hESCs and hiPSCs was similar up to 48 h. In contrast, at 96 h only hESCs increased glycolytic rate and up-regulated the TCA cycle with increased aspartate whereas hiPSCs down-regulated glutaminolysis and aspartate production. Hence, not only was the metabolism of ROCK-exposed hPSCs different from that of the day 0 control, but cells sequentially down-regulated metabolism at 12 and 24 hours, followed by an upregulated metabolic profile at 48 hours and had completely disparate physiology by 96 hours of exposure.

### Caspase-3 expression did not differ up to 48 hours of ROCK exposure

**Figure 4:**
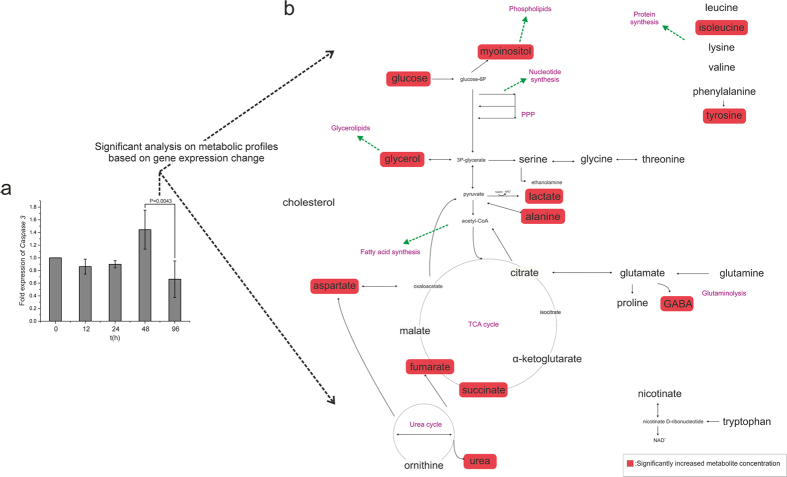
*Caspase 3* expression over time in hESCs. (**a**) *Caspase 3* expression is significantly reduced at 96 h compared with that at 48 h, (p < 0.05) (expressions always relative to 0 h). (**b**) This effect at 96 h is highlighted by the increased metabolite pools, as expected when cells commit to apoptosis, a metabolically-demanding process.

ROCK inhibition blocks *caspase-3* apoptotic signalling^31^,^32^. qRT-PCR analysis confirmed that there was no change in *caspase-3* expression at any time-point in the hiPSCs cultures (data not shown). In contrast, there was a significant decrease in *caspase-3* expression in hESCs from 48 h to 96 h of ROCK inhibition (Fig. 4a). This alteration of *caspase-3* from 48 h to 96 h may reflect on the increased activity of metabolic pathways such as glycolysis, TCA cycle, glycerolipids and phospholipids synthesis in hESCs, which is required for apoptosis^33^, as shown in Fig. 4b, after significant analysis and comparison of the metabolic profiles of the two time-points. Regardless, changes in metabolism observed throughout in the cultures of both hPSC types cannot be explained by changes in *caspase-3* expression, which remained stable for at least 48 hours and similar to that of 0 h control unexposed cells. In all cases, the viability of the cells remained high (data not shown).

### mTORC1 expression correlated with metabolic changes, independent of tp53

**Figure 5:**
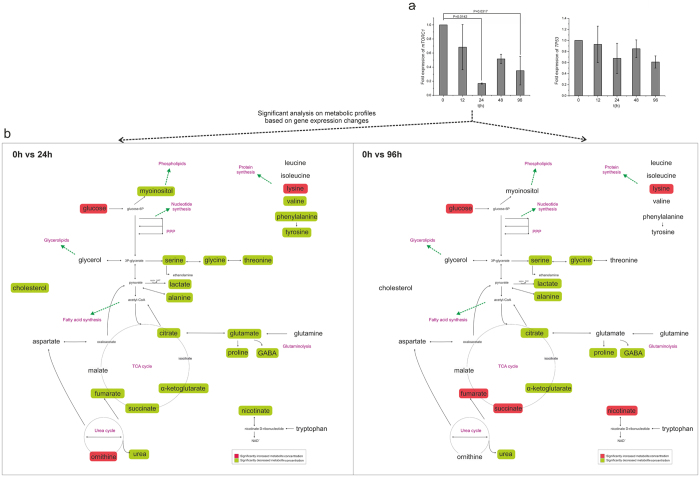
Expression of *mTORC1* and *tp53* in hESCs exposed to ROCK inhibitor and on-network representation. (**a**) *mTORC1* expression is significantly reduced at 24 h and 96 h compared with that at 0 h in hESCs (p < 0.05), whereas expression of *tp53* is unchanged throughout culture (expressions always relative to 0 h). (**b**) Reduction of *mTORC1* expression correlated with significant decreases in the concentration of metabolite pools at 24 h compared to 0 h and a further decrease at 96 h. The comparisons applied were chosen accordingly to the significant changes on gene expression.

**Figure 6:**
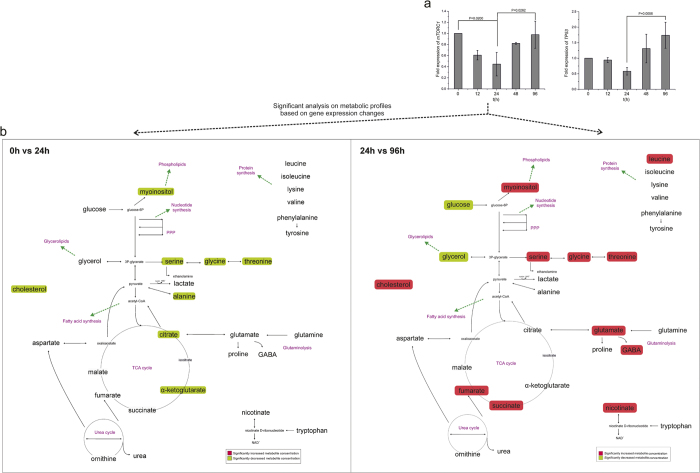
Expression of *mTORC1* and *tp53* in hiPSCs exposed to ROCK inhibitor and on-network representation. (**a**) *mTORC1* is less expressed at 24 h compared with that at 0 h; expression increases again at 96 h (p < 0.05). Expression of *tp53* is also increased at 96 h compared with that at 24 h (p < 0.05) (expressions always relative to 0 h). (**b**) These changes in *mTORC1* and *tp53* expression are reflected in the metabolic transitions, wherein metabolite pools decreased at 24 h and increased again at 96 h. The comparisons applied were chosen accordingly to the significant changes on gene expression.

Expression of two major metabolic controllers, *mTORC1* and *tp53*, was assessed. In hESC cultures inhibited by ROCK (Fig. 5), no difference in *tp53* expression throughout the culture time was observed. In contrast, *mTORC1* expression was lower at 24 h and 96 h exposure compared to the control 0 h unexposed cells (p < 0.05; Fig. 5a), correlating with decreased metabolic activity at the same time points (Fig. 5b), as identified by the significant analysis of the metabolic profiles between these time-points. Specifically, at 24 h glycolysis and glutaminolysis, lipid synthesis, most of the amino acid pools and TCA cycle were less active, whereas at 96 h glycolysis and glutaminolysis were reduced compared to the control 0 h unexposed group. In hiPSC cultures inhibited by ROCK (Fig. 6), expression of *tp53* was higher only at 96 h exposure compared with that at 24 h exposure, coincident with the increased glycolysis and glutaminolysis, TCA cycle and lipid synthesis at 96 h exposure (Fig. 6a and b). In contrast*, mTORC1* expression was lower following 24 h exposure and then increased again at 96 h exposure (p < 0.05), correlating with similar changes in metabolism. Therefore, in both hESCs and hiPSCs, decreased expression of *mTORC1* correlated with reduced metabolism after 24 h of exposure with Y-27632, independent of *tp53* and *caspase3* expression levels.

### ROS/RNS levels and catalase expression suggest a correlation with proliferative metabolism

The measurement of reactive oxygen and nitrogen species (ROS/RNS) indicated a reduction in ROCK inhibitor-treated hESCs throughout the culture period. In contrast, following an initial reduction of ROS/RNS at 12 h and 48 h, an increase in ROS/RNS was observed at 96 h in hiPSCs (Supplementary Figure 2). *Catalase* expression was also significantly increased at 96 h in hiPSCs cultures compared to 12 h, 24 h and 48 h. No significant change has been observed in *glutamylcysteine synthetase* (*GCS*) expression in both hESC and hiPSC cultures whereas a significant decrease of *glutathione peroxidase 1* (*GPX-1*) expression was observed only at 24 h in hESCs (Supplementary Figure 3).

## Discussion

The use of the ROCK inhibitor on hPSCs has been shown not to affect their stemness^18^,^25^. In contrast, its effect on metabolism has not been investigated. Herein, we confirm that treatment of hiPSCs and hESCs with ROCK inhibitor does not affect the expression of pluripotency markers at gene and protein level, whereas their metabolism is altered and becomes distinctly different. The observed metabolic changes appeared to be irreversible since the cells did not revert back to the metabolic signature of untreated cells. Furthermore, the metabolic transitions, in both cell types, were similar up to 48 h of ROCK inhibitor exposure and correlated with expression levels of *mTORC1* at 24 h of ROCK inhibition, but were independent of *tp53* or *caspase-3* expression. Finally, the detected metabolic differences between hESCs and hiPSCs indicate that although the two hPSC types share many phenotypic similarities, they are not physiologically identical suggesting that their metabolic features should be considered in designing hPSC bioprocesses to deliver robust maintenance and differentiation protocols.

The effects on metabolism could be explained by the single cell culture conditions that result in loss of cell-to-cell contact, changes to nutrient availability in single cell cultures compared to that for cells grown within colonies, and the time-period required for cells to adapt to an environment conducive to single cell culture. hPSCs are highly proliferative cells and share metabolic characteristics with cancer cells^5^,^26^,^27^. In tumors, the glycolytic rate is reduced when E-cadherin is unbound^34^ similar to what we observed when the 0 h-untreated cells cultured in colonies were compared with treated cells at other time-points. After 12 h and 24 h of exposure to ROCK inhibitor, the metabolism of both hESCs and hiPSCs was down-regulated, less glycolytic and, therefore, less proliferative. According to literature on mammalian signalling, an indirect connection between ROCK and mTORC1 exists. ROCK inhibition has been shown to dramatically suppress phosphorylation and activity of FAK (Focal Adhesion Kinase)^35^,^36^. FAK activation has also been correlated with increased proliferation via regulation of the Akt/mTORC1 pathway^37^. Recently, this relationship was demonstrated at the metabolic level, where FAK activation increased glycolytic metabolism (aerobic glycolysis), which substituted mitochondrial respiration^38^. Consequently, ROCK inhibition could lead to down regulated FAK activity followed by suppressed mTORC1 signaling and a decreased proliferative (glycolytic) metabolism, which is in agreement with our results, as early as 12 h.

The serine-glycine-threonine pathway is directly connected to one-carbon metabolism, which is the folate and methionine cycle, as independent modules. One-carbon metabolism cycles carbon units, amino acids, to maintain redox balance and promote biosynthesis. Folate cycle activation is a sign of nucleic acid biosynthesis^39^, among others. Studies in cancer metabolism have suggested that pathway activation is correlated with proliferation of cells^40^,^41^. Serine biosynthesis and glycine metabolism promote tumorigenesis^41^. Our results show that the serine-glycine-threonine pathway is down-regulated following 12 h and 24 h exposure and up-regulated after 48 h exposure for both hESCs and hiPSCs (Fig. 3). Overall, it is clear that the cells lose their highly proliferative physiology after the exposure to ROCK inhibitor and the dissociation into single cells as indicated by their metabolic profiles and the metabolic pathways affected. Their metabolic physiology never returned to the initial condition.

Metabolic transitions following 48 h exposure to ROCK inhibitor were similar for both hESCs and hiPSCs. In contrast, 96 h exposure resulted in divergent metabolism for the two cell types. In hESCs metabolism increased; in contrast, hiPSCs had decreased metabolic activity. hESCs and hiPSCs share similar but not identical metabolism^26^. This fact could explain why the same manipulation (i.e. dissociation of colonies and exposure to Y-27632) could cause different effect(s), especially when the treatment is prolonged, as was observed following 96 h exposure ROCK inhibitor. Both hESCs and hiPSCs reduced their metabolic activity trying to adapt to the new environment, something which is clear from the12 h and 24 h metabolic profiles. After 48 h exposure, the cells appear to finally adapt to the new environment and increase their metabolic activity again, assuming a more proliferative metabolism. The two cell types follow discrete directions afterwards. Moreover, even if gene and protein expression of pluripotency markers remained high following 96 h exposure to ROCK inhibitor, a small but significant decrease in the percentage of pluripotent cells was observed. This could explain the divergent effects on the metabolism between the two types of cells after 96 h of exposure, where hESCs increase their metabolite pools further while hiPSCs show a decrease in their metabolic activity when compared to 48 h exposure. It becomes clear that hESCs respond differentially to the prolonged exposure to ROCK inhibitor and single cultivation than hiPSCs.

Both hESCs and hiPSCs encounter a critical point in culture at 24 h of exposure, as indicated by the metabolic change commensurate with reduction in *mTORC1*. It has been shown that metabolism plays a significant role in defining human pluripotent stem cell fate, mostly by affecting the epigenome, in an irreversible way following a critical point^5^,^42^,^43^. Increased *mTORC1* expression favors growth metabolism, i.e. increased glycolysis and glutaminolysis, whereas the opposite effect is expected when *mTORC1* is not expressed. Interestingly, *tp53* expression is also increased at 96 h compared to 24 h in hiPSCs – this may represent an attempt of cells to control proliferative activity. High *mTORC1* expression^44^ induces *tp53*, as the two proteins counter-balance each other^45^,^46^, with *mTORC1* favoring growth when environmental conditions (nutrients, O_2_, etc.) are appropriate^47^, while *tp53* suppresses growth and proliferation when necessary^28^,^29^,^48^. ROCK inhibition blocks the pathway of myosin II to caspase-induced apoptosis^49^. In hESCs, *caspase-3* expression decreased after 96 h exposure but not at 48 h exposure, coincident with activation of the glycolysis pathway at 96 h with respect to production of glycerolipids and phospholipids, consistent with previous work that showed that caspase-driven apoptosis is metabolically demanding^33^.

ROS/RNS and *catalase* expression analyses indicate a positive correlation between proliferative metabolism and oxidative stress. The decrease in ROS/RNS levels in hESCs is followed by a decrease in *mTORC1* expression, while the increase in ROS/RNS levels and *catalase* expression at 96 h at hiPSCs cultures correlates with a more proliferative metabolism and increased *mTORC1* expression. Our results are in agreement with the literature. Specifically, a positive relationship between pluripotency and hPSC maintenance with proliferative metabolism and reduction of ROS^50^ has already been established. Similarly, ROCK inhibition has been linked to down-regulation of oxidative stress^51^.

Metabolism represents the cellular function that is most sensitive to even small environmental disturbances; GC-MS metabolomics has already been proven sensitive enough to detect changes in the physiology of cell cultures compared to gene and protein expression^52^, something of great value in the fields of stem cell biology and cell culture engineering in terms of monitoring and bioprocess optimization. Ultimately, metabolomics could be used as a release assay for verifying both product (cells) and bioprocess quality.

In conclusion, exposure to ROCK inhibitor altered cellular metabolism whereas gene and protein expression of pluripotency markers remained unaffected; as early as 12 h exposure to ROCK inhibitor resulted in the metabolism of both hESCs and hiPSCs being different than that of unexposed cells. Generally, hPSC metabolism decreased for the first 24 h of exposure and then increased again at 48 h exposure for both hESCs and hiPSCs, with completely disparate metabolic pathways followed following 96 h exposure. The observed metabolic changes correlated well with similar changes in *mTORC1* expression, a principal metabolic regulator, yet were independent of *tp53* and *caspase-3* expression levels. Our findings indicate that metabolomics is essential in deciphering changes of physiology and is an invaluable tool in the field of stem cell bioprocessing as it can be used to identify optimal physiological transitions and result in robust cultivation processes.

## Methods

### Cell Cultures

The pluripotent stem cell lines, hESCs (WA09) and hiPSCs (MR90-1) from WiCell Research Institute, WI, USA were used at passages 29 to 33. Cells were cultured in mTeSR1 (STEMCELL Technologies Canada Inc., Vancouver, BC) in Matrigel Matrix (Corning, NY, US) as recommended by the manufacturer. The hESCs and hiPSCs were either treated with 10 μM of Y-27632 ROCK inhibitor or not. Samples were collected at different time-points, 0-untreated, 12, 24, 48, 96 hours (h) and tested over the expression of pluripotency markers (Fig. 1A). The earliest time point that meaningful analysis could be performed is 12 h as the single cells need sufficient time to attach to the culture surface. Samples treated with ROCK inhibitor (Y-27632) where cultured in mTeSR1 with 10 μΜ Y-27632 (dihydrochloride - Tocris Bioscience, Bristol, UK) for 2 hours. Thereafter, cells were detached with the use of Accutase (StemPro - Thermo Fisher Scientific, MA, USA), colonies were dissociated into single cells by pipetting^22^,^23^,^24^, seeded at 30–40 * 10^4^ cells/cm^2^ density on Matrigel and cultured in mTeSR1 with 10 μΜ Y-27632 up to 96 h. Samples from three independent experiments (N = 3) were collected.

### Quantitative PCR

Total RNA from hESCs and hiPSCs was extracted using the RNeasy mini kit (QIAGEN Sciences, MD, USA). The primers used were: *sox2* F-5′-GCCGAGTGGAAACTTTTGTCG-3′, R-5′-GCAGCGTGTACTTATCCTTCTT-3′ 53, *oct4* F-5′-GTTGATCCTCGGACCTGGCTA-3′ R-5′-GGTTGCCTCTCACTCGGTTCT-3′ 53, *nanog* F-5′-GTCTTCTGCTGAGATGCCTCACA-3′ R-5′-CTTCTGCGTCACACCATTGCTAT-3′ 53, *caspase3* F-5′-AATTGTGGAATTGATGCGTGATG-3′ R-5′-CTACAACGATCCCCTCTGAAAAA-3′ 54, *tp53* F-5′-CCAGGGCAGCTACGGTTTC-3′ R-5′-CTCCGTCATGTGCTGTGACTG-3′ 55, *mtorc1* F-5′-CTGGGACTCAAATGTGTGCAGTTC-3′ R-5′-GAACAATAGGGTGAATGATCCGGG-3′ 56, *gapdh* F-5′-ACCACAGTCCATGCCATCAC-3′ R-5′-TCCACCACCCTGTTGCTGTA-3′ 56, F-5′-CCTTCTGGCACAGCACGTTG-3′ R-5′-TAAGACGGCATCTCGCTCCT-3′ *gcs*^57^, F-5′-CCTCAAGTACGTCCGACCTG-3 R-5′-CAATGTCGTTGCGGCACACC-3′ *gpx-1*^*60*^, F-5′-GCAGATACCTGTGAACTGTC-3′ R-5′-GTAGAATGTCCGCACCTGAG-3′ *catalase*^57^. One-step cDNA synthesis and PCR analysis was applied, using the KAPA SYBR FAST ABI Prism One-Step qRT-PCR kit (Kapa Biosystems, Inc. MA, US). Relative quantification was obtained measuring SYBR Green I fluorescence (ROX reference dye) with the StepOnePlus (Applied Biosystems - Thermo Fisher Scientific, MA, USA) qPCR instrument. Per reaction, 1 ng of RNA was used.

### Metabolite profiling

Cells were washed twice with PBS before cold methanol (−40 °C) was added to culture plates (6-well - Corning, NY, US). At least 10^6^ cells were extracted from each replicate-sample of hPSCs using ribitol (1 mg/1 × 10^6^ cells) as an internal standard, as previously described^52^. The process of methoximation (50 μL of 20 mg methoxyamine hydrochloride/mL pyridine) and MSTFA (100 μL of N-methyl-trimethylsilyl-trifluoroacetamide) derivatization turned the dried polar extracts into their (MeOx) TMS-derivatives^58^,^59^. Metabolic profiles were obtained using QP2010 Ultra GC-MS and GCMSsolution software Ver.4.11 (Shimadzu Corp., Kyoto, Japan). The raw metabolomics dataset was comprised of 84 peaks of known chemical category metabolites. From each independent sample, three experimental replicates (n = 3) were created. The relative peak areas of all detected peaks (RPAs) were estimated from their normalization with the 103 marker ion peak area of the internal standard ribitol. Data normalization and filtering are applied before incorporation into the final dataset table^58^,^59^. Cumulative (effective) peak areas were calculated using weight coefficients of the hESCs 0 h-untreated cells. All analyses applied on the acquired metabolic profiles were based on standardized values of the metabolite relative peak areas^52^.

### Flow Cytometry

Cells were assessed for both intracellular and extracellular markers of pluripotency. After fixation and permeabilization for intracellular marker expression, cells were stained with –phycoerythrin (PE)-conjugated Mouse anti-Human Nanog (Clone N31-355), PerCP-Cy5.5-conjugated Mouse anti-Oct3/4 (Clone 40/Oct3) and Alexa Fluor 647-conjugated Mouse anti-Sox2 (Clone 245610) (BD Human Pluripotent Stem Cell Transcription Factor Analysis Kit). For extracellular marker expression, cells were stained with phycoerythrin (PE)-conjugated -Rat anti-SSEA-3 (Clone MC631) and Alexa Fluor 647-conjugated Mouse anti-Human TRA-1-81 (Clone TRA-1-81) (BD StemFlow Human Pluripotent Stem Cells Sorting and Analysis Kit). Isotypes controls used were phycoerythrin (PE)-conjugated Rat IgM (R4-22), Alexa Fluor 647-conjugated Mouse IgM (G155-228), phycoerythrin (PE)-conjugated Mouse IgG (MOPC-21), PerCP-Cy5.5-conjugated Mouse IgG (X40), Alexa Fluor 647-conjugated Mouse IgG2a (MOPC-173). In all cases, cells were collected with the use of Accutase and counted using a standard haemocytometer. All antibodies were used at a concentration of 20 μL/sample obtained from BD Biosciences (BD Biosciences, San Jose, CA). BD LSRFortessa (BD Biosciences, San Jose, CA) was the instrument used for the analyses and 10000 events were analysed for each sample. FlowJo 10.1 (FlowJo LLC, OR, USA) software was used for the data analyses.

### Statistical analyses

One-way ANOVA with Bonferroni *post hoc* test was used for flow cytometry and qRT-PCR data comparison (N = 3, n = 3). The level of statistical significance was set by p < 0.05. The p-values where differences were significant are clearly noted on the figures. Unsupervised HCL and PCA algorithms were used as visualization methods of the differences among samples, based on their metabolic profiles (N = 3, n = 3). The Euclidean distance metric was used in HCL. SAM was used to identify the metabolites, whose concentration was significantly higher or lower in a set of metabolic profiles compared to another, with false discovery rate (FDR) at zero^60^. TM4 MeV v4.9.1 (Dana-Farber Cancer Institute, MA) was used for the multivariate^61^ and Origin2016 (OriginLab Corporation, MA) for univariate statistical analyses.

### Immunocytochemistry

Sox2 and Oct4 protein expression was assessed using immunofluorescence. Cells were washed with PBS and fixed with 4% (w/v) paraformaldehyde solution in PBS (Sigma-Aldrich, UK) and subsequently blocked with a PBS solution of 10% (v/v) normal donkey serum, 0.1% (w/v) Triton X-100 and 1% (w/v) BSA (Sigma-Aldrich, UK) for 45 min at room temperature in order to avoid non-specific antibody binding. After blocking, the cells were incubated for 3 h with conjugated antibodies at 1:10 dilution (NL557 PE-conjugated Goat Anti-Human SOX2, NL637 Alexa Fluor 647-conjugated Goat Anti-Human Oct-4) (R&D Systems Inc, MN) at room temperature and counterstained with DAPI for 5 min. Images were taken with a BX-51 Olympus microscope (Olympus, UK).

### ROS/RNS assay

Reactive oxygen species (ROS) and reactive nitrogen species (RNS) levels were evaluted with the use of quenched fluorogenic probe dichlorodihydrofluorescin (DCFH-DiOxyQ; OxiSelect, Cell Biolabs, CA). Cells were resuspendend at a cell density of 1 × 10^7^ cells/mL in PBS and lysed with 1% (w/v) Triton X-100 solution and spinned at 10000 g for 5 min to remove insoluble particles. They were assayed according to the manufacturer’s instructions.

## Additional Information

**How to cite this article**: Vernardis, S. I. *et al*. Human embryonic and induced pluripotent stem cells maintain phenotype but alter their metabolism after exposure to ROCK inhibitor. *Sci. Rep.* **7**, 42138; doi: 10.1038/srep42138 (2017).

**Publisher's note:** Springer Nature remains neutral with regard to jurisdictional claims in published maps and institutional affiliations.

## Supplementary Material

Supplementary File

Supplementary Table
