# Supplementary material for: Human embryonic and induced pluripotent stem cells maintain phenotype but alter their metabolism after exposure to ROCK inhibitor
Source: Sci Rep. 2017 Feb 6;7:42138. doi: 10.1038/srep42138 (PMC5292706; doi:10.1038/srep42138)
Supplement: Supplementary File [file srep42138-s1.pdf]

Human embryonic and induced pluripotent stem cells maintain phenotype but alter their metabolism after exposure to ROCK inhibitor

Spyros I. Vernardis[1], Konstantinos Terzoudis[1], Nicki Panoskaltsis[1,2], Athanasios Mantalaris[1,*]

[1]Biological Systems Engineering Laboratory, Department of Chemical Engineering, Imperial College London, UK

[2]Department of Haematology, Imperial College London

Supplementary information

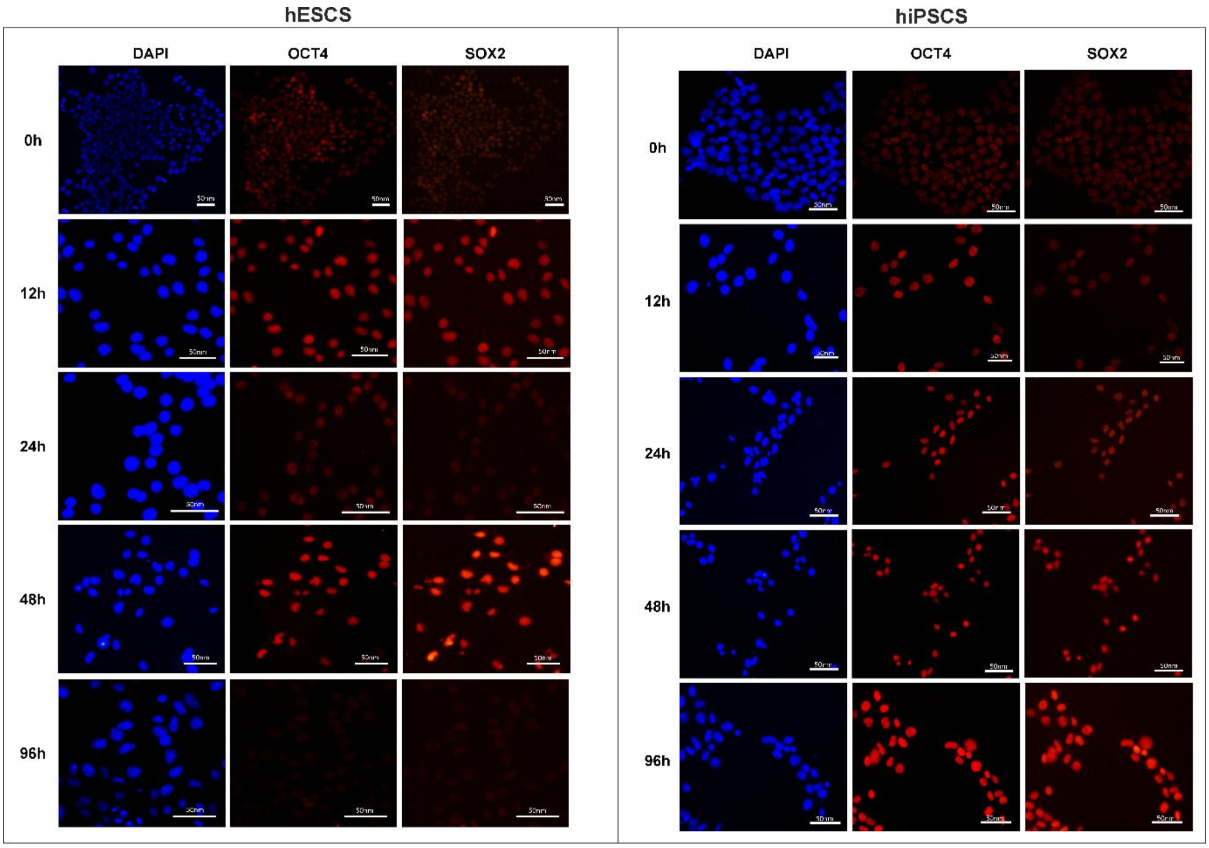

Supplementary Figure 1: Immunostaining for pluripotency markers (OCT4, SOX2). The stemness is maintained in high levels after treatment with ROCK inhibitor and cultivation of the cells as singles. A loss of pluripotency can be detected at 96h in hESC cultures.

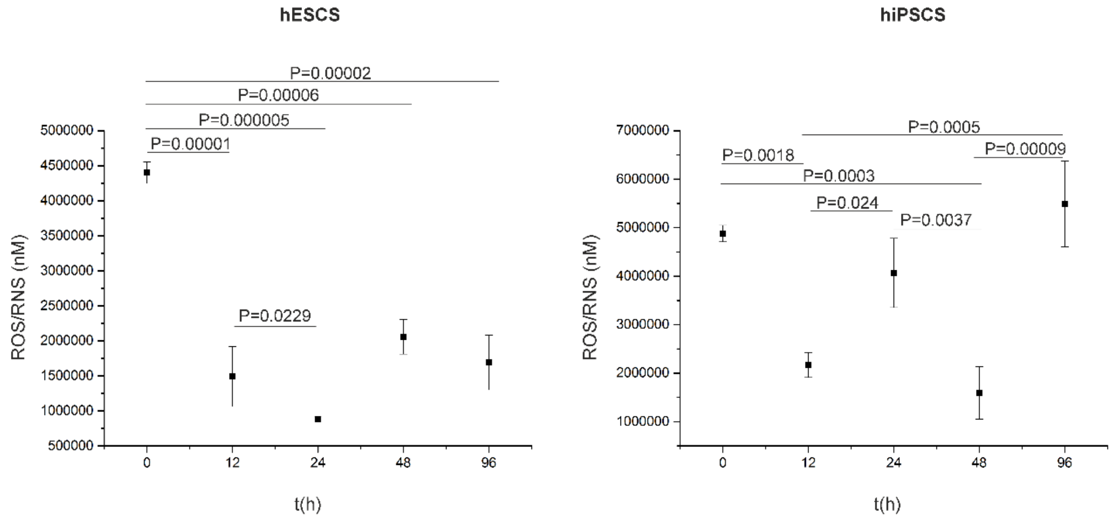

Supplementary Figure 2: ROS/RNS measurements of hESC and hiPSC cultures. A significant decrease in free radicals is observed after treatment with ROCK inhibitor in hESCs cultures. In hiPSCs there is an increase in 48h compared to 24h and a high increase in 96h.

hESCs **hiPSCs**

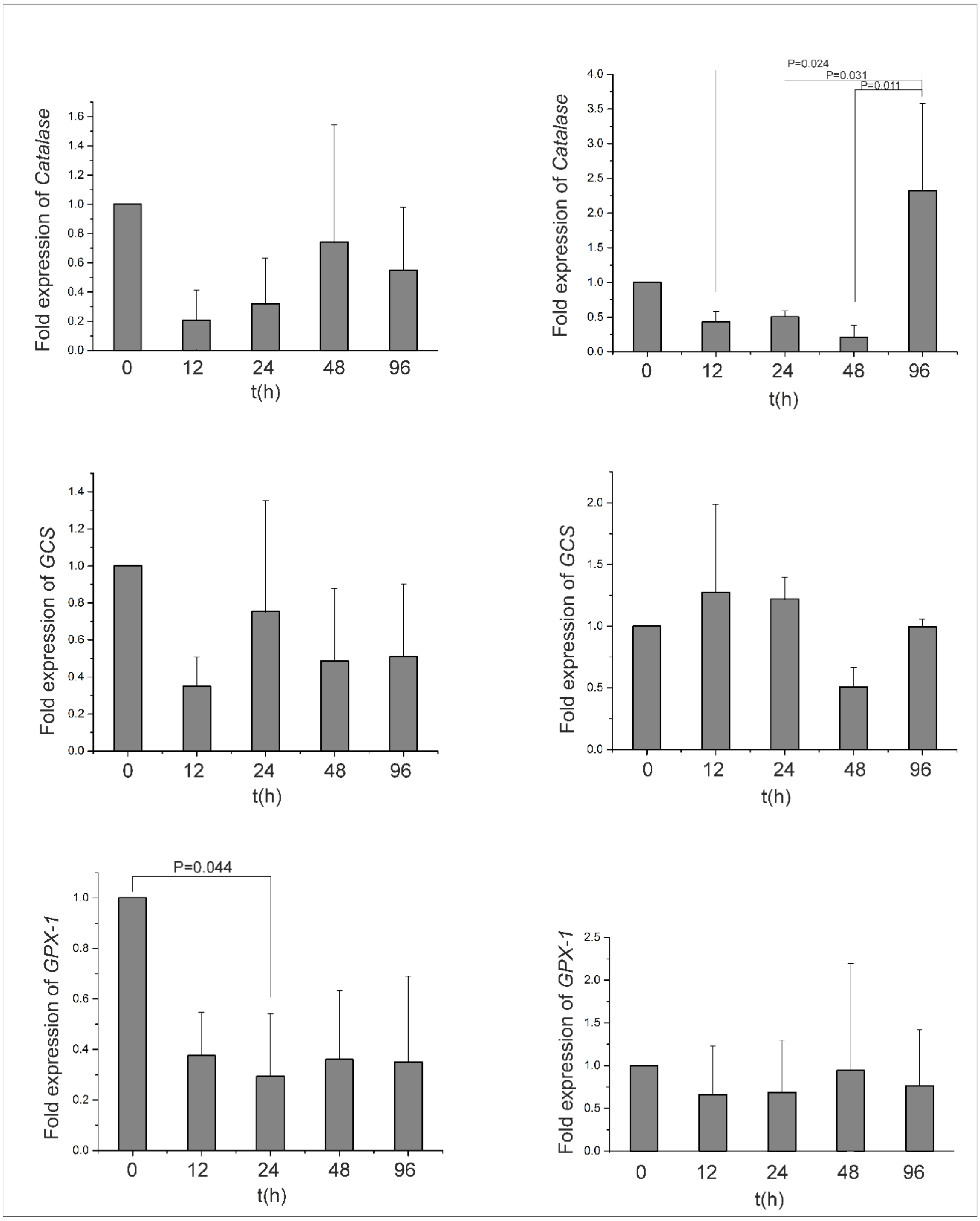

<u>Supplementary Figure 3:</u> Antioxidant enzymes expression on hESC and hiPSC cultures. A significant increase in *catalase* expression is observed in hiPSCs at 96h compared to 12h, 24h and 48h. *GPX-1* is significantly downregulated at 24h compared to 0h.

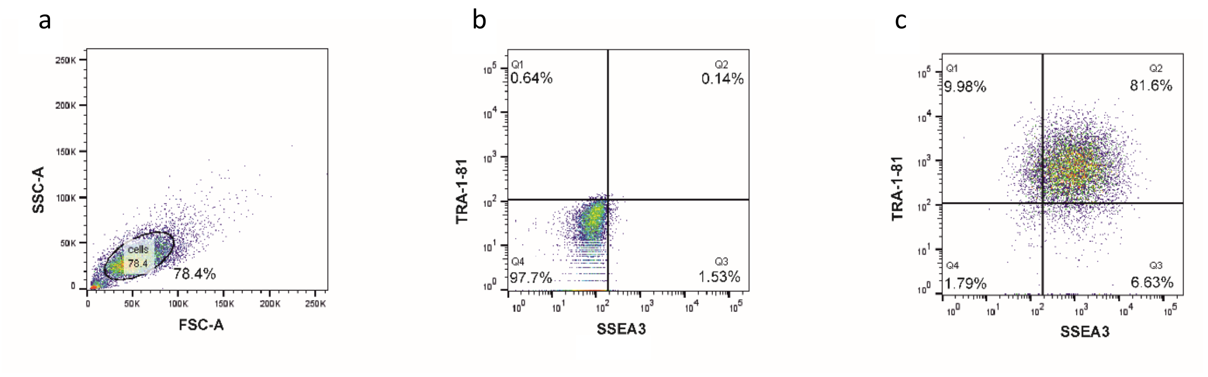

Supplementary Figure 4: Gating strategy for extracellular markers of pluripotency. a) cell population gating, b) isotype control for hiPSCs 12h, c) sample for hiPSCs 12h. (The axes are linear and percentages of the numbers of the cells are shown in each graph section)

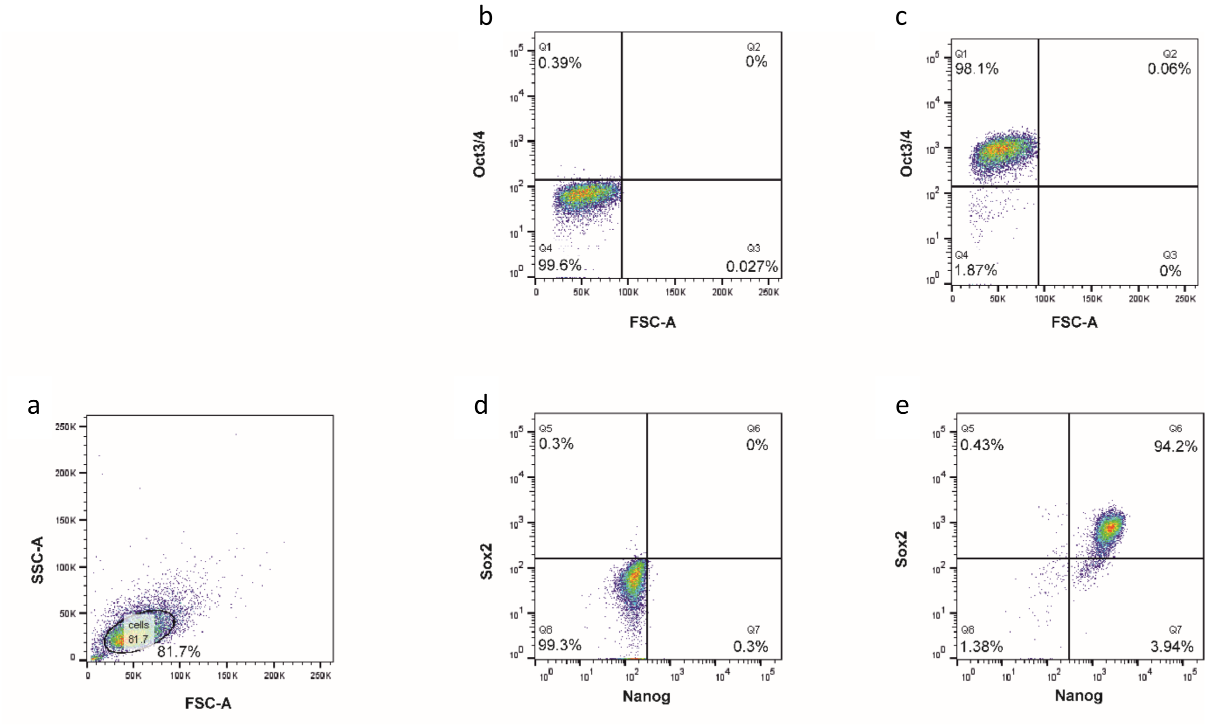

Supplementary Figure 5: Gating strategy for intracellular markers of pluripotency. a) cell population gating, b) isotype control of OCT3/4 for hiPSCs 12h, c) sample of OCT3/4 for hiPSCs 12h, d) isotype control SOX2 / NANOG out of OCT3/4 gating for hiPSCs 12h, e) sample SOX2 / NANOG out of OCT3/4 gating for hiPSCs 12h. (The axes are linear and percentages of the numbers of the cells are shown in each graph section)
