# Supplementary material for: Human embryonic and induced pluripotent stem cells maintain phenotype but alter their metabolism after exposure to ROCK inhibitor
Source: Sci Rep. 2017 Feb 6;7:42138. doi: 10.1038/srep42138 (PMC5292706; doi:10.1038/srep42138)
Supplement: Supplementary Table [file srep42138-s2.pdf]

Human embryonic and induced pluripotent stem cells maintain phenotype but alter their metabolism after exposure to ROCK inhibitor

Spyros I. Vernardis[1], Konstantinos Terzoudis[1], Nicki Panoskaltsis[1,2], Athanasios Mantalaris[1]

[1]Biological Systems Engineering Laboratory, Department of Chemical Engineering, Imperial College London, UK

[2]Department of Haematology, Imperial College London

Supplementary information

SUPPLEMENTARY FILE: The metabolite peaks detected in the MS-reconstructed chromatograms

PEAK NAME	METABOLITE	Marker Ion	Retention time (RT)	Chemical Category (Note 1)	P/NQ (Note 2)	[illegible]
α-ketoglutarate	α-ketoglutarate	112	24.845	1	P	[illegible]
alanine 2tms	alanine	116	7.815	3	P	[illegible]
asparagine 5tms	asparagine	116	27.905	3	P	[illegible]
aspartate 2tms	aspartate	160	19.82	3	P	[illegible]
aspartate 3tms	aspartate	232	20.66	3	P	[illegible]
benzoic acid 1tms	benzoic acid	179	15.395	1	P	[illegible]
cholesterol	cholesterol	129	50.48	1	P	[illegible]
citrate 4tms	citrate	273	27.38	1	P	[illegible]
fumarate 2tms	fumarate	245	16.18	1	P	[illegible]
gaba 2tms	gaba	102	14.446	3	NQ	[illegible]
gaba 3tms	gaba	174	19.925	3	P	[illegible]
glucopyranose (peak 1)	glucopyranose	204	27.245	2	P	[illegible]
glucopyranose (peak 2)	glucopyranose	204	29.045	2	P	[illegible]
Glucose Meox 1	Glucose	319	27.127	2	P	[illegible]
Glucose Meox 2	Glucose	319	27.42	2	P	[illegible]
glutamate 2tms	glutamate	84	22.75	3	P	[illegible]
glutamate 3tms	glutamate	246	23.095	3	P	[illegible]
glutamine 4tms	glutamine	156	23.49	3	P	[illegible]
glycerol 3tms	glycerol	205	12.1	1	P	[illegible]
glycine 3tms	glycine	248	13.59	3	P	[illegible]
hexadecanoic acid 1tms	hexadecanoic acid	117	31.73	1	P	[illegible]
iso-citrate 4 tms	iso-citrate	273	27.795	1	NQ	[illegible]
isoleucine 2tms	isoleucine	86	11.23	3	P	[illegible]
isoleucine 3tms	isoleucine	158	13.435	3	P	[illegible]
lactate 2tms	lactate	117	7.03	1	P	[illegible]
leucine 1tms	leucine	131	10.81	3	P	[illegible]
leucine 2tms	leucine	158	12.735	3	P	[illegible]
lysine 5tms	lysine	156	31.73	3	P	[illegible]
maltose	maltose	204	40.83	2	NQ	[illegible]
methionine 2tms	methionine	128	21.515	3	P	[illegible]
methyl benzoate 1tms	methyl benzoate	193	18.725	1	P	[illegible]
monomethyl phosphate	monomethyl phosphate	241	13.48	1	P	[illegible]
myo-inositol 6tms	myo-inositol	305	29.855	1	P	[illegible]
octadecanoic acid	octadecanoic acid	117	35.25	1	P	[illegible]
ornithine	ornithine	70	24.435	3	P	[illegible]
phenylalanine 2 tms	phenylalanine	218	24.25	3	P	[illegible]
phenylalanine O tms	phenylalanine	120	24.545	3	P	[illegible]
phosphoric acid 4tms*	phosphoric acid	299	14.446	1	P	[illegible]
proline 1tms	proline	142	14.49	3	P	[illegible]
pyruvate	pyruvate	174	9.39	1	NQ	[illegible]
serine 2tms	serine	116	14.32	3	P	[illegible]
serine 3tms	serine	204	15.57	3	NQ	[illegible]
succinate 2tms	succinate	247	16.335	1	P	[illegible]
threonine 2tms	threonine	130	14.93	3	P	[illegible]
threonine 3tms	threonine	291	15.99	3	NQ	[illegible]
tyrosine NOO	tyrosine	218	30.435	3	P	[illegible]
urea	urea	189	15.06	1	P	[illegible]
valine 1tms	valine	72	9.185	3	P	[illegible]
valine 2tms	valine	144	11.04	3	P	[illegible]
un_1		159	4.279	1	NQ	[illegible]
un_10		103	15.115	2	P	[illegible]
un_11		111	15.315	1	NQ	[illegible]
un_12		105	15.405	1	P	[illegible]
un_13		117	16.325	1	P	[illegible]
un_17		86	18.975	3	NQ	[illegible]
un_18		88	20.42	1	P	[illegible]
un_19		103	20.75	2	P	[illegible]
un_20		104	20.845	1	NQ	[illegible]
un_21		100	21.67	1	NQ	[illegible]
un_22		100	24.225	1	P	[illegible]
un_24		116	25.335	3	P	[illegible]
un_26		103	26.555	2	NQ	[illegible]
un_27		156	27.67	3	NQ	[illegible]
un_3		91	4.435	1	NQ	[illegible]
un_30		205	29.205	1	NQ	[illegible]
un_31		105	51.71	1	P	[illegible]
un_32		103	40.32	2	NQ	[illegible]
un_34		110	12.275	1	P	[illegible]
un_37		89	13.925	1	P	[illegible]
un_39		115	5.79	1	NQ	[illegible]
un_38		110	14.05	1	P	[illegible]
un_41		128	6.37	3	P	[illegible]
un_43		131	7.03	1	P	[illegible]
un_45		152	7.995	1	P	[illegible]
un_46		130	8.355	1	P	[illegible]
un_47		89	8.655	1	P	[illegible]
un_5		170	4.895	1	NQ	[illegible]
un_8		84	25.28	1	P	[illegible]
un_9		117	43.575	1	P	[illegible]
ethanolamine	ethanolamine	174	11.082	3	P	[illegible]
glycerate	glycerate	292	14.900	1	NQ	[illegible]
nicotinate	nicotinate	245	16.100	1	P	[illegible]
aminomalonic acid	aminomalonic acid	218	20.000	1	P	[illegible]
ethanolamine phosphate	ethanolamine phosphate	299	26.200	1	NQ	[illegible]
ribitol (Internal Standard)		103	22.535			[illegible]

Note 1: Chemical category 1, 2 and 3 is used as defined in Kanani and Klapa, 2007; Category 10 and 11 has been assigned to peaks that are considered artifacts due to column bleeding; 3(1) is assigned to category 3 metabolites that form only one derivative; 1(IS) is assigned to Internal Standard compound (in this case ribitol) which is a category 1 metabolite; 2(IS) is assigned to internal standard compound (in this case [U-13C] glucose which is a category 2 metabolite

Note 2: A peak is defined P or NQ, respectively, if it has beed quantified in at least one of the acquired metabolic profiles (P),or it has been detected in at least one of the acquired metabolic profiles but not in a quantifiable amount (and/or due to the shape of the peak)

PEAK NAME	METABOLITE	Marker Ion	Retention time (RT)	Chemical Category (Note 1)	P/NQ (Note 2)	hipscs0_a_Inj1	hipscs0_a_Inj2	hipscs0_a_Inj3
un_1		159	4.279	1	NQ	0	0	0
un_3		91	4.435	1	NQ	0	0	0
un_5		170	4.895	1	NQ	0	0	0
un_39		115	5.79	1	NQ	0	0	0
un_41		128	6.37	3	P	491104	393492	264813
lactate 2tms	lactate	117	7.03	1	P	18569115	20535433	21634138
un_43		131	7.03	1	P	2815066	2966025	3093265
alanine 2tms	alanine	116	7.815	3	P	13191897	11948841	10560910
un_45		152	7.995	1	P	49824	38284	57197
un_46		130	8.355	1	P	19989	15136	16774
un_47		89	8.655	1	P	67333	61519	96721
valine 1tms	valine	72	9.185	3	P	3608937	3289108	4040567
pyruvate	pyruvate	174	9.39	1	NQ	0	0	0
leucine 1tms	leucine	131	10.81	3	P	187409	178706	189023
valine 2tms	valine	144	11.04	3	P	759002	631236	472657
ethanolamine	ethanolamine	174	11.082	3	P	749511	677352	708282
isoleucine 2tms	isoleucine	86	11.23	3	P	2453462	2217563	2613971
glycerol 3tms	glycerol	205	12.1	1	P	193667	145369	160806
un_34		110	12.275	1	P	3592433	3173919	3599309
leucine 2tms	leucine	158	12.735	3	P	227645	198527	151772
isoleucine 3tms	isoleucine	158	13.435	3	P	310204	281152	223881
monomethyl phosphate	monomethyl phosphate	241	13.48	1	P	643032	510305	552936
glycine 3tms	glycine	248	13.59	3	P	3702506	3215972	3580777
un_37		89	13.925	1	P	105133	90719	99486
un_38		110	14.05	1	P	1038400	1281625	3943583
serine 2tms	serine	116	14.32	3	P	2137630	2073013	2350538
gaba 2tms	gaba	102	14.446	3	NQ	0	0	0
phosphoric acid 4tms*	phosphoric acid	299	14.446	1	P	0	0	0
proline 1tms	proline	142	14.49	3	P	6177406	4662406	3381495
glycerate	glycerate	292	14.900	1	NQ	624	0	464
threonine 2tms	threonine	130	14.93	3	P	782864	785853	896397
urea	urea	189	15.06	1	P	173000	139209	133268
un_10		103	15.115	2	P	634925	511522	557002
un_11		111	15.315	1	NQ	0	0	0
benzoic acid 1tms	benzoic acid	179	15.395	1	P	83224	71231	77395
un_12		105	15.405	1	P	507235	433424	475793
serine 3tms	serine	204	15.57	3	NQ	0	0	0
threonine 3tms	threonine	291	15.99	3	NQ	0	0	0
nicotinate	nicotinate	245	16.100	1	P	33878	26236	31214
fumarate 2tms	fumarate	245	16.18	1	P	33878	26236	31751
un_13		117	16.325	1	P	29910	35071	33599
succinate 2tms	succinate	247	16.335	1	P	17395	14359	17278
methyl benzoate 1tms	methyl benzoate	193	18.725	1	P	393832	333774	352693
un_17		86	18.975	3	NQ	0	0	0
aspartate 2tms	aspartate	160	19.82	3	P	24597	23414	23205
gaba 3tms	gaba	174	19.925	3	P	45537391	41993363	43322113
aminomalonic acid	aminomalonic acid	218	20.000	1	P	514066	469599	18319
un_18		88	20.42	1	P	694062	627226	726070
aspartate 3tms	aspartate	232	20.66	3	P	101887	119509	123892
un_19		103	20.75	2	P	111102	105877	106324
un_20		104	20.845	1	NQ	0	0	0
methionine 2tms	methionine	128	21.515	3	P	34884	41968	40427
un_21		100	21.67	1	NQ	0	0	0
glutamate 2tms	glutamate	84	22.75	3	P	8493613	7040884	7523291
glutamate 3tms	glutamate	246	23.095	3	P	20949721	15619786	15934879
glutamine 4tms	glutamine	156	23.49	3	P	9278311	7090309	7393904
un_22		100	24.225	1	P	555894	522128	552154
phenylalanine 2 tms	phenylalanine	218	24.25	3	P	204628	198177	212038
ornithine	ornithine	70	24.435	3	P	94707	77555	80398
phenylalanine 0 tms	phenylalanine	120	24.545	3	P	171992	125437	137927
α-ketoglutarate	α-ketoglutarate	112	24.845	1	P	66360	77714	63276
un_8		84	25.28	1	P	454081	358403	377405
un_24		116	25.335	3	P	226734	528691	650007
ethanolamine phosphate	ethanolamine phosphate	299	26.200	1	NQ	740841	842325	639340
un_26		103	26.555	2	NQ	0	0	0
Glucose Meox 1	Glucose	319	27.127	2	P	1422297	897587	1013966
glucopyranose (peak 1)	glucopyranose	204	27.245	2	P	560204	472013	438181
citrate 4tms	citrate	273	27.38	1	P	116825	106585	121360
Glucose Meox 2	Glucose	319	27.42	2	P	55805	42874	53739
un_27		156	27.67	3	NQ	0	0	0
iso-citrate 4 tms	iso-citrate	273	27.795	1	NQ	0	0	0
asparagine 5tms	asparagine	116	27.905	3	P	170936	186565	217000
glucopyranose (peak 2)	glucopyranose	204	29.045	2	P	1216442	988955	1085915
un_30		205	29.205	1	NQ	0	0	0
myo-inositol 6tms	myo-inositol	305	29.855	1	P	3961100	2967289	3078076
tyrosine NOO	tyrosine	218	30.435	3	P	232610	468445	474886
hexadecanoic acid 1tms	hexadecanoic acid	117	31.73	1	P	14156349	12915841	13399488
lysine 5tms	lysine	156	31.73	3	P	10590	6294	10211
octadecanoic acid	octadecanoic acid	117	35.25	1	P	10557343	9064527	363319
un_32		103	40.32	2	NQ	0	0	0
maltose	maltose	204	40.83	2	NQ	0	0	0
un_9		117	43.575	1	P	291853	144866	201264
cholesterol	cholesterol	129	50.48	1	P	3333496	3298297	3446804
un_31		105	51.71	1	P	970348	715521	690488
ribitol (Internal Standard)		103	22.535			6713387	5963824	2841734

Note 1: Chemical category 1, 2 and 3 is used as defined in Kanani and Klapa, 2007; Category 10 and 11 has been assigned to peaks that are considered artifacts due to column bleeding; 3(1) is assigned to category 3 metabolites that form only one derivative;
1(IS) is assigned to Internal Standard compound (in this case ribitol) which is a category 1 metabolite; 2(IS) is assigned to internal standard compound (in this case [U-13C] glucose which is a category 2 metabolite
